# Effects of Dietary Lipid Levels on Growth Performance, Hematological Parameters, and Muscle Fatty Acid Composition of Juvenile *Arapaima gigas*

**DOI:** 10.3390/ani15142027

**Published:** 2025-07-10

**Authors:** Carlos Andre Amaringo Cortegano, Luz Angélica Panaifo-García, Nidia Llapapasca, Nieves Sandoval, Adhemir Valera, Juan Rondón Espinoza, Gonzalo Orihuela, Andrea Carhuallanqui, Daphne D. Ramos-Delgado, Fred W. Chu-Koo, Ligia Uribe Gonçalves

**Affiliations:** 1 Facultad de Medicina Veterinaria, Universidad Nacional Mayor de San Marcos, Lima 15001, Peru; langelicapanaifog@gmail.com (L.A.P.-G.); nllapapascag@unmsm.edu.pe (N.L.); nsandovalc@unmsm.edu.pe (N.S.); avaleraa@unmsm.edu.pe (A.V.); jrondone@unmsm.edu.pe (J.R.E.); gonzalo.orihuela@unmsm.edu.pe (G.O.); andrea.carhuallanqui@unmsm.edu.pe (A.C.); dramosd@unmsm.edu.pe (D.D.R.-D.); 2 Programa de Pós-Graduação em Ciência Animal e Recursos Pesqueiros, Universidade Federal do Amazonas, Manaus 69067-005, AM, Brazil; 3 Facultad de Ciencias, Universidad Nacional Autónoma de Alto Amazonas–UNAAA, Yurimaguas 16501, Peru; fchu@unaaa.edu.pe; 4 Grupo de Investigación en Nutrición y Genómica Nutricional de Peces Amazónicos-NUGENUPA, Universidad Nacional Autónoma de Alto Amazonas–UNAAA, Yurimaguas 16501, Peru; 5 Departamento de Zootecnia, Faculdade de Zootecnia e Engenharia de Alimentos, Universidade de São Paulo, Pirassununga 13635-900, SP, Brazil

**Keywords:** carnivorous fish, fish farming, lipid requirement, pirarucu, weight gain

## Abstract

This study evaluates the effects of dietary lipid levels on growth performance, hematological health, and muscle composition of juvenile *Arapaima gigas*. We tested five isonitrogenous diets (451.7 g kg^−1^ of crude protein) with increasing lipid levels (6%, 10%, 14%, 18%, and 22%). A total of 600 juvenile *A. gigas* (80.0 ± 10.5 g; 21.8 ± 1.0 cm) were distributed into 20 tanks (500 L; *n* = 4; 30 fish per tank) in an indoor open system. The fish were fed to apparent satiety four times daily for 60 days. As dietary lipid levels increased, all growth parameters and lipid content in both the whole body and muscle declined. The diet containing 6% lipids resulted in the maximum final weight, weight gain, feed intake, and the lowest feed conversion rate. However, a maximum lipid level of up to 10.26%, with a gross energy-to-protein ratio of 10.15 kcal g^−1^ in the diet, as determined through polynomial regression analysis, can be used for juvenile *A. gigas* without significantly affecting weight gain. Diets with high lipid content (18% and 22% lipids) resulted in the lowest survival rates, highest feed conversion rates, lowest condition factor, visible skeletal protrusions, scale depigmentation, and impaired blood biochemistry. The content of eicosapentaenoic acid, docosahexaenoic acid, n-3, and the n-3:n-6 ratio increased in the muscle lipid fraction (mg g^−1^ of total lipids) in response to higher dietary lipid levels; however, this does not represent an overall improvement in the meat quality, since the total lipid content in the muscle (g of lipid per 100 g of muscle) was reduced due to impaired growth in fish fed high-lipid diets. Notably, the experimental diets also differed in fatty acid composition, which may have influenced some of the physiological and compositional responses observed. Diets with 6% lipids are recommended to provide optimal growth performance, and a maximum dietary lipid level of up to 10.26% is advised to ensure successful *A. gigas* farming without impairing weight gain.

## Simple Summary

This study investigates how increasing dietary lipid levels affect the growth performance, hematological health, and muscle quality of juvenile *Arapaima gigas*, a large fish native to the Amazon. Five diets with lipid levels ranging from 6% to 22% were tested. After 60 days of feeding, the fish fed with a 6% lipid diet showed the best growth. It was also found that lipid levels up to 10.26% did not significantly affect weight gain. However, higher lipid levels (18% and 22%) led to lower survival rates, poorer growth performance, and other health issues. These findings can help improve diet formulation for *A. gigas*, leading to better growth performance, healthier fish and higher-quality meat.

## 1. Introduction

Aquaculture has established itself as one of the most important producers of animal protein globally, and its sustained development depends on the optimization of farming practices and aquafeed formulations that maximize the production and health of the farmed species [1]. *Arapaima gigas* (Schinz, 1822), commonly known as “paiche” in Peru or “pirarucu” in Brazil and Colombia, it is the world’s largest scaled freshwater fish and is native to the Amazon basin. It belongs to the most basal branch of extant Teleostei [2] and its farming is on the rise in Amazonian countries; moreover, this species has already been introduced in the United States, China, Cuba, Mexico, the Philippines, Singapore, and Thailand [3] as a promising alternative for food security. Due to its large size, taste and nutritional meat quality [3,4], its filet is sold in Peruvian and Brazilian markets at various prices, ranging from USD 7.0 to 12.0 per kg, and *A. gigas* represents a potential food resource not only for regional trade in Amazonian regions, but also for national and international trade [3]. As interest in *A. gigas* increases in aquaculture, it is essential to understand its nutritional requirements and the impact of nutrition on its productivity, health, and meat quality.

Dietary lipids are one of the key components in aquafeed formulation, as they play a fundamental role in fish growth and health, as well as in the quality of the final product [5,6,7]. This is particularly relevant given that fish are the primary natural source of long-chain omega-3 fatty acid, especially eicosapentaenoic acid (20:5n-3; EPA) and docosahexaenoic acid (22:6n-3; DHA), which are essential for human health and increasingly scarce in global diets [5,6]. Lipids provide an elevated amount of energy and supply essential fatty acids for cell function, hormone synthesis, and cell membrane formation [6]. Adequate dietary lipid levels can improve the diet’s palatability, resulting in higher feed intake and, therefore, better fish growth performance [5]. Nonetheless, excessive lipids and energy can lead to adverse effects in fish, such as excessive accumulation of fat in tissues, health problems, damage to liver functions, reduction in growth performance, increased mortality, and a decrease in the final product quality [8,9,10].

Determining the optimal range of lipid levels in diets for carnivorous fish has gained attention in aquaculture because of its capacity to improve feed conversion rate, increase protein conversion rates, reduce nitrogen excretion and promote better growth, and the poor capacity to digest carbohydrates as energy sources in carnivorous fish [9,11]. It is considered that diets containing between 10 and 20% of dry weight as lipids promote efficient protein use for growth performance, without excessive fat accumulation in the tissues [12,13]. However, it depends on different conditions such as the species, age and the system production, as determined for *Rachycentron canadum*—5.70% lipids [14], *Pseudoplatystoma coruscans*—18% lipids [15], as well as for *Oncorhynchus mykiss* and *Salmo salar*, where diets containing up to 24% and 40% lipids, respectively, have promoted better performance and a protein-sparing effect [16].

Although *A. gigas* is not considered a significant source of EPA and DHA compared to other freshwater and marine species [17,18,19], determining the optimal range of dietary lipid levels for this species remains essential for enhancing nutritional management strategies and improving production efficiency. Therefore, this study aims to evaluate the effects of different dietary lipid levels on growth performance, hematological health, body composition, and fatty acid content in juvenile *A. gigas*, since there is no scientific information available regarding dietary lipid levels for this species and it is known that *A. gigas* has limited ability to digest dietary carbohydrates as an energy source [20].

## 2. Materials and Methods

This study was approved by the Ethical Committee of Animal Experimentation and Research of the Faculty of Veterinary Medicine of the Universidad Nacional Mayor de San Marcos, Lima, Peru (Protocol No. 2023-04).

### 2.1. Experimental Diets

Five isonitrogenous diets (451.7 g kg^−1^ of crude protein—CP) were formulated [21,22] using the same protein ingredients to contain increasing levels of lipids: 6%, 10%, 14%, 18%, and 22% (6DL, 10DL, 14DL, 18DL, and 22DL) and, consequently, increasing gross energy (GE) content and the GE:CP ratio (9.7; 10.1; 10.6; 10.9; 11.4 kcal g^−1^). The lipid content of the diets considered the lipid content determined for all the ingredients used in the formulation (Table 1). All the dried ingredients were ground (1000 µm; McFord FFC-15), homogenized in a horizontal mixer (Pial MPI-100, Pucallpa, Peru) for 20 min, and extruded using a 4 mm diameter pellet matrix in a twin screw extruder (Shandong, FT75P, Taishan, China). The pellets were dried in a continuous flow tubular dryer for 25 min (90 °C) until they reached a moisture content of less than 10% (Biobase, BM-50-1, Jinan, China). While the pellets were still warm after drying, the required amount of fish oil to achieve lipid levels between 10% and 22% was sprayed over the feeds using a pump. The buoyancy of the pellets was then observed over a period of 10 min in water, and all pellets remained afloat, indicating good physical integrity. Finally, the diets were packaged and stored in opaque bags in a freezer at −15 °C. Aliquots of the diets for weekly use were kept in opaque, air-sealed, off-white plastic bottles to prevent oxidation.

The dietary lipid levels (6–22%) were selected to represent a progressive range of lipid inclusion, covering from minimal levels found in commercial juvenile diets (6%) to upper thresholds typically used in high-energy feeds for large carnivorous fish (up to 22%). These values were adapted from previous studies in tropical carnivorous species such as *P. corruscans* and *R. canadum* [14,15].

### 2.2. Feeding Trial

The feeding trial was carried out at the Aquaculture Experimental Station of IVITA of the Universidad Nacional Mayor de San Marcos (8°38′32.9″ S, 74°57′06.8″ W), Pucallpa, Peru. All the fish came from the same spawning and were obtained from a local fish farm. Prior to starting the experiment, we confirmed the absence of endoparasites and ectoparasites by euthanizing 15 fish via brain puncture [23] to ensure the sanitary safety.

After acclimation, fish were randomly selected and assigned to tanks. Each dietary treatment was randomly distributed across four replicate tanks (n = 4), and all tanks were maintained under identical environmental and management conditions to minimize tank effects. In that sense, juvenile *A. gigas* (80.0 ± 10.5 g; 21.8 ± 1.0 cm) were randomly distributed into twenty fiberglass tanks (500 L; 30 fish per tank) [24], housed in an indoor open system using water from an artesian well with constant water renovation (flow rate: 1.5 L min^−1^) and a 12 h photoperiod. All the tanks were siphoned once daily to remove fish feces. The experiment followed a completely randomized design with five treatments (diets) and four replications each (fish tanks; n = 4). The water temperature (27.7 ± 0.5 °C), dissolved oxygen (6.3 ± 0.3 mg L^−1^), and pH (7.2 ± 0.2) were measured once daily at 7 a.m., using a multiparameter (HANNA, HI9819, Woonsocket, RI, USA). Total ammonia (1.4 ± 0.2 mg L^−1^) and nitrite (0.7 ± 0.2 mg L^−1^) were monitored twice weekly using colorimetric kits (LaMotte, 3533-05, Chestertown, MD, USA) [25,26]. The water quality parameters remained within the comfort range for *A. gigas* during the entire study [27]. The fish were fed the experimental diets in four meals daily (8 a.m., 12 p.m., 4 p.m., and 8 p.m.) to apparent satiety for 60 days. Feed was offered gradually in multiple passes per feeding time to ensure consumption. Although commercial farming of *A. gigas* generally involves two or three daily meals, in this experimental trial, fish were fed four times daily to maximize feed intake, ensure homogeneity among individuals, and reduce potential dominance or competition. This approach is common in laboratory-based nutritional studies with juvenile fish to increase intake accuracy and minimize environmental bias. As *A. gigas* is a fast-growing fish, in the biometry on day 30, ten fish were removed from each tank (the five smallest fish and the five largest fish) to ensure the adjustment of asymmetry among the fish in each experimental unit, in addition to providing an adequate density for growth, and avoiding dominance, thus ensuring their welfare, as proposed by Epifânio et al. [28].

At the end of the feeding trial, all the fish in each tank were measured and the growth performance was obtained via the following calculations: survival (%) = (final number of animals × 100)/initial number of animals; final weight (g); final length (cm); weight gain (g) = final weight − initial weight (wet weight basis); length gain (cm) = final length−initial length; feed intake (g) = feed offered/number of fish; feed conversion rate (rate) = feed intake/weight gain (both on a wet weight basis); protein conversion rate (%) = ((final body weight g × final whole-body protein %) (initial body weight g × initial whole-body protein %)/protein consumed g) × 100 (both on a dry weight basis); relative growth rate (% day^−1^) = (e^g^ − 1) × 100, e = Euler’s Number, g = [(ln final weight − ln initial weight)/experimental time]; and Fulton’s allometric condition factor (ratio) = final weight/final length^3^.

From each tank, three fish were randomly selected to be photographed in order to describe the body condition [29]. Two fish were destined for blood collection by puncturing the caudal vessel with heparinized syringes. Then, ten fish were euthanized by thermal shock in cold water [30] and all of them were used for somatic index analysis [hepatosomatic index = liver weight/final fish weight × 100 (both in wet weight basis); and lipo-somatic index = viscera fat weight/final fish weight × 100 (both in wet weight basis)]; from those fish, two were used for whole-body composition analysis (a sample pool per tank) and two fish were used for muscle composition analysis (a sample pool per tank).

### 2.3. Whole-Blood and Plasma Analysis

The hemoglobin concentration was determined using the cyanometahemoglobin method [31], using Drabkin’s reagent, with the absorbance read at 540 nm using a spectrophotometer (Unico, 1205, Dayton, NJ, USA). Hematocrit was determined using the Goldenfarb et al. [32] scale. Erythrocytes were counted in a Neubauer chamber under an optical microscope with a 40× objective (10 μL of blood, 2.0 mL of citrate formaldehyde) (3Scientific, Y J-2005B, Ningbo, China). Corpuscular constants were determined using the methods described by Brown [33] and the following calculations: mean corpuscular hemoglobin concentration (%) = [hemoglobin] × 100/hematocrit; mean corpuscular volume (fL) = hematocrit × 10/erythrocytes; and mean corpuscular hemoglobin (g dL^−1^) = [hemoglobin] × 10/erythrocytes. Total leukocytes were counted via an indirect method [34] in blood smears and underwent May–Grunwald–Giemsa staining [35]. Approximately 500 erythrocytes of each smear were counted, as were the number of leukocytes. The total numbers of leukocytes and thrombocytes were estimated using the ratio of the number of total erythrocytes (obtained in the Neubauer chamber) according to the formula: total leukocytes (μL) = number of leukocytes × number of erythrocytes_Neubauer chamber_ (μL)/number of erythrocytes_slide_ [34].

Determination of glucose (mg dL^−1^), triglycerides (mg dL^−1^), cholesterol (mg dL^−1^), and total protein (g dL^−1^) from plasma were performed after whole blood centrifugation (4 °C, 12,000 rpm per 180 s), using commercial kits and spectrophotometric (Unico, 1205, Dayton, NJ, USA) readings [28,36].

### 2.4. Proximate Composition of Diets and Fish

Proximate composition analyses were carried out in triplicate for each sample. The proximate composition of the diets, whole body, and muscle of the fish were determined according to the AOAC [37] at the Natura Analitica SAC Laboratory, Pucallpa, Peru.

### 2.5. Fatty Acid Composition Diets and Muscle

Fatty acid content analyses were carried out in triplicate for each sample. Total lipids of the diets and muscle samples were determined using the Bligh and Dryer [38] method. For the fatty acid analyses of the diets and samples of muscle, fatty acid methyl esters (FAME) were prepared using the method proposed by Santos-Júnior et al. [39]. The methyl esters were separated using gas chromatography (Pekin-Elmer AutoSystem XL USA Gas Chromatographer, Waltham, MA, USA), fitted with a flame ionization detector (FID) and a fused-silica capillary column (SUPELCOWAX^TM^10, Darmstadt, Germany) of 30 m in length, 0.25 mm internal diameter, and 0.25 μm film thickness. The operation parameters were as follows: temperature detector, 270 °C; injection port temperature, 250 °C; column temperature, 180 °C for 30 min, ramp rate of 2 °C min^−1^ up to 250 °C, with a final holding time of 14.5 min; carrier gas, hydrogen at 1.2 mL min^−1^; nitrogen was used as the make-up gas at 30 mL min^−1^; and split injection at a ratio of 1:80. For identification, the retention times of the fatty acids were compared to those of standard methyl esters (Sigma, St. Louis, MO, USA). Retention times and peak area percentages were automatically computed using the Software TotalChrom Workstation 6.0 (Waltham, MA, USA). Quantification of the fatty acids (mg g^−1^ of total lipids) was performed using tricosylic acid (23:0) methyl ester (Sigma-Aldrich, St. Louis, MO, USA) as an internal standard [40]. Theoretical FID correction factor values were used to obtain concentration values of fatty acids in mg g^−1^ of total lipids [41] using the equation below: FA = [(AX × WIS × CFX)/(AIS × CFAE × WX)]; in which FA is a fatty acid in mg g^−1^ of total lipids, AX is the peak area (fatty acids), WIS is the standard weight (mg), CFX is the theoretical correction factor, AIS is the standard peak area (23:0), CFAE is the necessary conversion factor in order to express results in mg of fatty acid rather than as methyl ester, and WX is the sample weight (g). The fatty acid analysis was performed in the LABS of the Instituto Tecnológico de la Producción, Lima, Peru.

### 2.6. Data Analysis

The homogeneity of the initial weights of the fish was tested using Cochran’s Q test (*p* < 0.05). Growth performance data, hematological parameters, proximate composition, and fatty acid composition were analyzed using one-way ANOVA (*p* < 0.05) after confirming normality by Shapiro–Wilk test (*p* < 0.05) and homoscedasticity via the Barlett test (*p* < 0.05). When significant differences were detected (*p* < 0.05), Tukey’s multiple comparison test was used to determine differences among treatment means. The optimal dietary lipid level to maximize growth performance and the highest lipid level that does not impair the growth performance in juvenile *A. gigas* were determined through polynomial regression analysis. The selection of the regression model was based on the data goodness-of-fit coefficient of determination R^2^ and Akaike’s information criterion for the overall model and, among models of the same subset, the F Test was used. The data were processed using Statistica Software 13.0 [42].

## 3. Results

### 3.1. Fish Growth Performance

All the growth performance parameters in juvenile *A. gigas* were affected by the increasing levels of dietary lipids (*p* < 0.05) (Table 2). During the first 30 days of feeding, no mortalities were observed; however, at the end of the trial, the lowest survival rates (86.3%) were recorded in fish fed 18DL and 22DL. The highest values for final weight, weight gain, final length, length gain, feed intake, protein conversion rate, and relative growth rate were observed in fish fed 6DL and 10DL, though decreased significantly in fish fed higher-lipid diets. Additionally, fish fed 6DL to 14DL presented better feed conversion rates and higher condition factor values, respectively, while these parameters were impaired in fish fed higher levels of dietary lipids.

**Figure 1:**
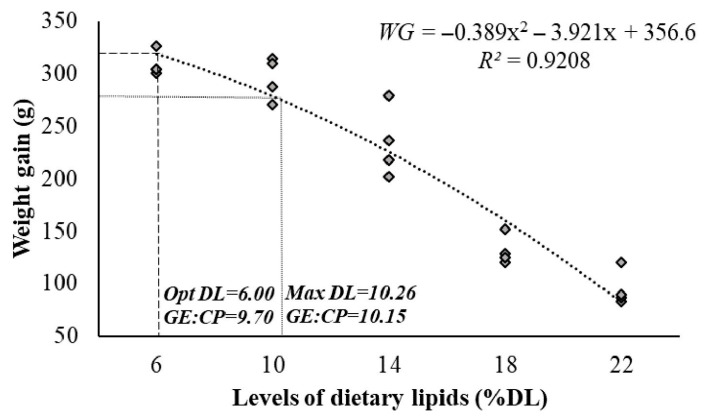
Effect of increasing dietary lipid levels on weight gain in juvenile *Arapaima gigas* over a 60-day experimental period. The dietary lipid level of 6%, corresponding to a gross energy-to-crude protein (GE:CP) ratio of 9.70 kcal g^−1^, was identified as optimal for maximizing weight gain. The maximum lipid level of 10.26%, corresponding to a GE:CP ratio of 10.15 kcal g^−1^, was defined as the threshold beyond which weight gain is significantly impaired, based on a second-order polynomial regression.

**Figure 2:**
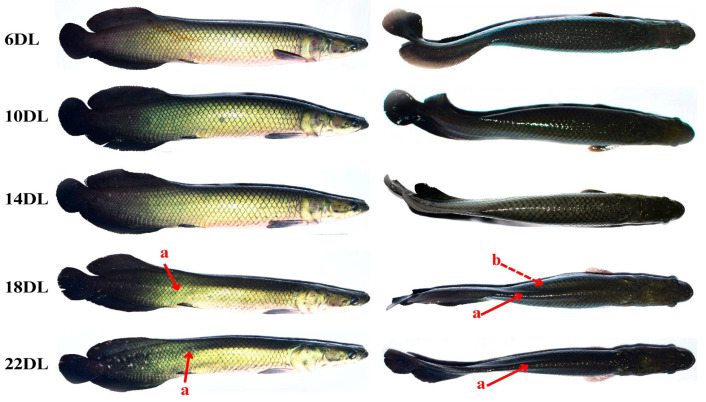
Body condition in juvenile *Arapaima gigas* fed diets with increasing levels of lipids (6%, 10%, 14%, 18%, and 22%) for 60 days. The red arrows indicate the following: a, prominent bone structures; b, depigmentation in the scales.

Data for the final weight, weight gain, feed conversion rate, and relative growth rate were fitted to a second-order polynomial regression. Therefore, 6DL supports the maximum final weight, weight gain, feed intake, and the best feed conversion rate in juvenile *A. gigas*. However, the maximum dietary lipid level should not exceed 10.26%, with a gross energy to protein ratio of 10.15 kcal g^−1^, as higher levels (14DL, 18DL, and 22DL) were found to impair weight gain (Figure 1). The values of the hepatosomatic and lipo-somatic indexes were not altered in any of the fish in this study (*p* > 0.05). The body condition of juvenile *A. gigas* was impaired by 18DL and 22DL, with prominent skeletal protrusions and depigmentation of the scales (Figure 2).

### 3.2. Whole-Blood and Plasma Analysis

The hemoglobin (7.9 ± 1.3 g dL^−1^), erythrocytes (2.6 ± 0.6 × 10^6^ dL^−1^), mean corpuscular volume (112.0 ± 20.6 fL), mean corpuscular hemoglobin (32.3 ± 7.8 g dL^−1^), leucocytes (0.1 ± 0.02 × 10^5^ µL^−1^), neutrophils (80.4 ± 8.3%), lymphocytes (19.2 ± 8.3%), triglycerides (42.8 ± 16.2 mg dL^−1^), and cholesterol (64.0 ± 20.7 mg dL^−1^) values in the juvenile *A. gigas* were not affected by the different levels of dietary lipids (*p* > 0.05). Fish fed 6DL presented the lowest hematocrit value and the highest mean corpuscular hemoglobin concentration (*p* < 0.05), while these parameters were constant in the fish fed 10DL to 22DL (*p* > 0.05). In fish fed up to 14DL, the plasma glucose content was similar (37.8 ± 12.2 mg dL^−1^) and higher than those reported in the fish fed 18DL and 22DL (22.9 ± 5.2 mg dL^−1^). The lowest levels of plasmatic protein were found in fish fed 18DL (Table 3).

### 3.3. Proximate Composition of the Whole Body and Muscle

Moisture, crude protein, and ash content in the whole body and the muscles of the fish were not affected by the different levels of dietary lipids tested (*p* > 0.05). Nonetheless, lipid content in both the whole body and muscle decreased in fish fed 14DL to 22DL and 18DL to 22DL, respectively, in comparison with lower-fat diets (*p* < 0.05) (Table 4).

### 3.4. Fatty Acid Composition of Muscle

The data are expressed in mg g^−1^ of lipids. Therefore, twenty-one fatty acids were identified in the muscle lipid fraction of the juvenile *A. gigas*, with polyunsaturated fatty acids (PUFAs) predominating, followed by saturated fatty acids (SFAs), in all the fish groups. Stearic acid (18:0) was the only fatty acid whose content remained unchanged (*p* > 0.05). In contrast, the content of long chain-PUFAs, such as EPA, DHA, and n-3, increased within the muscle lipid fraction in response to the increasing levels of dietary lipids. An inverse response was observed for all the other fatty acids as dietary lipid levels increased. Palmitic acid (16:0) and oleic acid (18:1n-9c) were the predominant acids in the SFA and monounsaturated fatty acid (MUFA) groups, respectively. Regarding PUFAs and long chain-PUFAs, linoleic acid (18:2n-6, LA), and DHA were the most abundant. However, the LA content was higher in fish fed lower-lipid diets, while DHA predominated in the fish fed higher-lipid diets, with both acids showing a decrease and increase, respectively, as the dietary lipid levels increased. Fish fed diets with higher levels of omega-3 fatty acids (14DL to 22DL), which contained progressively increasing concentrations of EPA and DHA, exhibited greater deposition of these fatty acids in the muscle, particularly DHA. In contrast, LA, which was most abundant in the 6DL and 10DL diets (169.9 and 148.4 mg g^−1^ of total lipid, respectively), was also deposited in higher concentrations in the muscle of fish fed these lower-lipid diets.

Despite this, the n-3 content was higher in the lipid fraction than the n-6 content in all fish groups, and the n-3 content directly influenced the n-3:n-6 ratio, showing higher values in the fish fed high-lipid diets (Table 5).

## 4. Discussion

Fish fed diets with lower lipid levels (6DL and 10DL) exhibited the best growth performance outcomes. In contrast, dietary lipid levels exceeding 14%, had detrimental effects on the growth performance of *A. gigas*. This suggests a limited protein-sparing effect from dietary lipids and/or a low energy–protein requirement for *A. gigas* [10]. Although lipids are better utilized than carbohydrates as a non-protein energy source for carnivorous fish and *A. gigas*, excess lipids increase the dietary energy content, which can reduce feed intake as fish quickly reach satiety [20,43], a pattern observed in the fish from the 14DL, 18DL, and 22DL groups. The insufficient feed intake may limit the supply of essential nutrients for fish, increasing the feed conversion rate and impairing growth performance, potentially hindering development or leading to weight loss [44,45,46]. Yin et al. [43] reported that excess dietary lipids contribute to the downregulated expression of the farnesoid X receptor, sterol 26-hydroxylase, and cholesterol 7 alpha-hydroxylase genes, disturbing lipids digestion in *Micropterus salmoides*, a freshwater carnivorous fish as *A. gigas*. The authors suggested that the expression of these genes promotes bile acid production, which may enhance lipid digestion and, consequently, improves growth performance.

Fish fed up to 14DL presented better values of feed conversion rate in comparison with the other fish groups. These feed conversion rate values were slightly higher than those obtained in other studies using juvenile *A. gigas* [4,21,36] in response to aquafeeds with adequate protein content and/or using feed additives. In the present experiment, it is possible that the well water used to supply the tanks influenced the slightly higher feed conversion rate values due to the presence of minerals or other factors in the water that could have affected the fish’s digestion and metabolism, as observed in previous studies by this research group [36]. Excess dietary energy is often associated with adverse effects on the overall fish health, leading to deteriorated body conditions that may manifest as prominent bone structures and depigmentation of the scales [9,10,44], as observed in the fish fed 18DL and 22DL. Typically, increased dietary lipid intake results to greater fat deposition [9,10]; however, these effects were not observed in our study. In addition, fish fed 18DL and 22DL reached satiety more quickly, resulting in lower feed intake and the deterioration in body condition, which may suggest a specific metabolic adjustment in *A. gigas* to dietary lipids, whereby lipids are not stored in fish muscle or viscera but are potentially utilized or eliminated through other pathways. Despite the negative effects observed with high dietary lipid levels, hepatosomatic and lipo-somatic indexes did not show significant differences among the groups of fish. The hepatosomatic and lipo-somatic indexes consider the accumulation of nutrients in the liver and visceral fat [6], respectively, relative to fish’s weight. Thus, proportionally, the fat-to-body weight ratio remained similar across groups.

The best growth performance in terms of final weight, weight gain, feed conversion, and relative growth rate was estimated using polynomial regression analysis for fish fed a diet with 6% lipids; however, no adverse effects on weight gain were estimated in fish fed diet with up to 10.26% lipids, with a gross energy to protein ratio of 10.15 kcal g^−1^. These results align with studies performed in other carnivorous fish such as *Oxyeleotris marmorata*—12.1% of lipids [45], *Epinephelus malabaricus*—about 9% of lipids [47], and *Psetta maxima*—about 13% of lipids [48], in which a maximum lipid level was recommended to avoid deteriorating the productivity and health status of the fish.

The dietary lipid levels did not significantly affect most of the hematological and plasma biochemical parameters evaluated. However, dietary lipid level affected the red blood cell concentration and size, since fish fed 6DL presented the lowest hematocrit value and the highest mean corpuscular hemoglobin concentration value, while these parameters remained constant in fish fed 10DL to 22DL. Hematocrit values recorded in the fish fed 10DL to 22DL are close to those previously reported in *A. gigas* farmed under different feeding strategies [36,49,50], but lower than those values reported for Nobre et al. [4] in a study with juvenile *A. gigas* fed DHA-rich diets, and do not necessarily reflect a compromised health status. Changes in the hematocrit value may be related to the capacity of the fish to adjust to different levels of nutritional stress, thus maintaining general homeostasis [51].

Fish fed 18DL and 22DL presented lower plasma glucose when compared to those fed up to 14DL. Excess of dietary lipids could affect the energy metabolism in *A gigas*, since high-lipid diets can compromise the bioconversion of fatty acids in the gluconeogenesis pathway, reduce the bioavailability of plasma glucose and, consequently, produce negative impacts on the energy available for the growth of the fish [9,10,43]. Likewise, the lowest plasma protein content was observed in the fish fed 18DL, which is possibly due to the reduction in protein synthesis or to the availability of essential amino acids for the fish from the diet, indicating lower efficiency in the utilization of high-lipid diets. The relationship between high levels of dietary lipids and the decrease in plasma proteins in fish may suggest insufficient amino acid intake due to an imbalance in the dietary energy-to-protein ratio, which could lead to the utilization of protein and amino acids in basal metabolic reactions rather than for muscle synthesis [9,43,44,47]. Similarly, the inhibition of voluntary intake may account for the lower whole-body lipid content in fish fed 14DL to 22DL; however, the lipid content in both whole body and muscle in all fish were higher than those reported in muscle of *A. gigas* fed DHA-rich diets [4], but lower than those reported from wild [3].

The predominance of the fatty acid groups in the experimental diets was not reflected in the muscle lipid fraction, since the diets presented a predominance of SFAs, followed by PUFAs. The increase in dietary lipids caused a decrease in the palmitic acid, SFAs, and MUFAs content in the muscle. The decrease in the palmitic acid content and, consequently, SFAs in the muscle of *A. gigas*, may be associated with differences in triacylglycerol (TAG) storage and utilization, as lower lipid diets are known to promote higher TAG deposition with increased SFA and MUFA content, while higher-lipid diets may result in reduced TAG accumulation and altered FA partitioning [18].

The total content of PUFAs, EPA, DHA, n-3, and the n-3:n-6 ratio increased in the muscle lipid fraction in response to the increase in dietary lipids and to the composition of their fatty acids. PUFAs have a tendency to be retained more efficiently in fish tissues, since they represent poor substrates for mitochondrial β-oxidation and, consequently, are less used for energy production [6,7]. However, this does not represent an overall improvement in the meat quality, since the total lipid content in the fish (g of lipid per 100 g of muscle) and the growth were impaired in those fed high-lipid diets.

In the literature, it is reported that LA and linolenic acid (18:3n-3; LNA) are recognized as essential for tropical freshwater fish [6]. Other freshwater carnivorous fish species, *O. mykiss* and *M. salmoides*, have nutritional requirements of 7.0 to 20.0 mg g^−1^ of total lipids for LNA and 5.0 to 16.6 mg g^−1^ of total lipids for LA [5,52]. Diets with 6% and 10% lipids in our study provided 8.7–11.7 mg g^−1^ of total lipids of LNA and 86.2–152.8 mg g^−1^ of LA, suggesting that these experimental diets meet essential fatty acid requirements for freshwater carnivorous fish like *A. gigas*. However, *A. gigas* presents distinct lipid profiles, with muscle FA composition richer in omega-6 PUFAs [3], *so that could be interpreted carefully*. While LA and LNA are essential, their long-chain derivatives such as arachidonic acid (20:4n-6; ARA), EPA, and DHA play critical physiological roles, particularly in eicosanoid synthesis, membrane function, and neural development. Carnivorous fish such as *A. gigas* may depend on dietary sources of these LC-PUFAs due to limited endogenous synthesis capacity [5,6,12].

The ARA was not detected in the diets; however, low measurable levels were found in the muscle of all fish groups. This suggests that *A. gigas* may synthesize ARA endogenously [3,6,12]. This de novo synthesis of ARA might imply an additional metabolic cost, potentially diverting energy from growth and other physiological processes [12]. Although wild *A. gigas* are characterized by a muscle fatty acid content richer in omega-6 PUFAs such as ARA [3], the choice to increase dietary lipids via fish oil in this study reflects standard practices in carnivorous fish aquaculture, where fish oil is widely available and used as a functional lipid source. The use of fish oil resulted in variations in the fatty acid content, particularly the progressive increase in the n-3:n-6 ratio, which could have influenced the physiological responses observed in *A. gigas*.

Although n-3 fatty acids have been reported as beneficial for fish growth and health, an overall imbalance in dietary n-3 ratio, due to excessive n-3, can negatively impact fish growth. For freshwater fish, the recommended level of n-3 is around 100 mg g^−1^ of total lipids, while the experimental diets with 14% to 22% lipids contained 173.0–200.9 mg g^−1^ of total lipids of n-3, 1.7–2.0 times the recommended amount, which, combined with the excess dietary energy, may have affected health and negatively impacted fish growth. Excessive dietary n-3 fatty acids in fish may lead to metabolic disruption, competition with other energy sources, lipid peroxidation causing cellular damage and oxidative stress, and a weakened immune response [53].

## 5. Conclusions

Fish fed high-energy diets reached apparent satiety without consuming sufficient essential nutrients, resulting in lower weight gain and affecting body and muscle lipid and fatty acid compositions. High dietary lipid levels can negatively affect the body condition and survival in juvenile *A. gigas* and can also negatively impact the blood biochemistry of fish, which may compromise their health and increase susceptibility to disease. The fatty acid content of the muscle was influenced more directly by the dietary fatty acid content than by total lipid content, highlighting the importance of formulating diets based not only on lipid levels but also on the balance and identity of key fatty acids. Diets with 6% lipids are recommended to provide optimal growth performance, while a maximum dietary lipid level of up to 10.26%, with a gross energy-to-protein ratio of 10.15 kcal g^−1^, is advised to ensure successful *A. gigas* farming without impairing weight gain.

## Figures and Tables

**Table 1:** Feedstuffs and the proximate and fatty acid compositions of the experimental diets with increasing levels of dietary lipids for juvenile *Arapaima gigas* (dry matter basis).

	Diets ^1^				
	6DL	10DL	14DL	18DL	22DL
Ingredients (g kg^−1^)					
Fishmeal ^2^	560.0	565.0	568.0	580.0	585.0
Soybean meal	180.0	180.0	180.0	180.0	180.0
Corn grain	156.0	110.0	65.0	12.0	0.0
Wheat flour	90.0	90.0	90.0	90.0	56.0
Fish oil ^3^	0.0	41.0	83.0	124.0	165.0
Premix ^4^	10.0	10.0	10.0	10.0	10.0
Antifungal ^5^	2.0	2.0	2.0	2.0	2.0
BHT ^6^	2.0	2.0	2.0	2.0	2.0
Proximate composition (g kg^−1^)					
Dry matter	945.1	926.2	927.0	955.1	951.6
Crude protein–CP	452.9	452.0	450.0	452.9	450.9
Gross energy–GE (kcal kg^−1^) ^7^	4406.0	4574.0	4756.1	4950.9	5137.7
Crude fiber	23.2	22.5	21.8	21.0	16.7
Ash	153.0	163.5	171.4	177.3	187.6
Carbohydrates ^8^	309.5	261.3	215.8	168.1	123.9
Total lipids	61.4	100.7	141.0	180.7	220.9
GE:CP (kcal g^−1^)	9.7	10.1	10.6	10.9	11.4
Fatty acid composition (mg g of total lipids^−1^)					
∑SFA ^9^	356.0	377.7	388.9	447.8	460.0
14:0	35.0	39.6	40.4	48.9	46.9
16:0	230.6	239.7	245.9	280.0	292.5
18:0	63.9	67.9	70.9	83.0	85.8
∑MUFA ^10^	283.2	281.1	264.3	260.0	241.5
18:1n-9c	197.6	182.6	178.3	174.5	170.9
∑PUFA ^11^	290.7	284.1	285.0	290.0	290.0
18:2n-6	152.8	86.2	70.9	46.6	43.4
18:3n-3	11.7	8.7	8.5	6.4	6.1
20:5n-3	28.5	32.3	33.9	42.0	42.9
22:6n-3	68.8	83.8	101.6	127.3	128.3
∑n-3	132.3	153.2	173.0	196.3	200.9
∑n-6	153.9	87.5	72.3	47.7	44.6
N-3:n-6	0.9	1.8	2.4	4.1	4.5

^1^ Means of triplicate analyses by sample (n = 3) for proximate composition and fatty acid content. ^2^ Premium fishmeal (COSTAMAR^®^, Chiclayo, Peru) derived from anchovies with 630.0 g kg^−1^ of crude protein, 4199.0 kcal kg^−1^ of gross energy, 92.1 g kg^−1^ of lipids, and 194.0 g kg^−1^ of ash. ^3^ Crude fish oil (COSTAMAR^®^) derived from anchovies. ^4^ Premix of vitamins and minerals (DSM AQUACULTURE, Piura, Peru)—DSM is a premix of ROVIMIX^®^ vitamins (Piura, Peru), MICROGRAN^®^ minerals (Piura, Peru), BHT, and BHA (antioxidants) for animal use, produced by DSM NUTRITIONAL PRODUCTS PERU, and contains (per kg of product): vitamin A 9 334,000.0 IU; vitamin D 3 1,866,800.0 IU; vitamin E 93,333.0 IU; vitamin K3 5.3 g; thiamine (B1) 12.0 g; riboflavin (B2) 13.3 g; pyridoxine (B6) 10.0 g; vitamin B12 0.02 g; ascorbic acid 210.0 g; niacin 100.0 g; pantothenic acid 33.3 g; folic acid 2.7 g; biotin 0.5 g; copper 1.0 g; iron 13.3 g; manganese 26.7 g; cobalt 0.1 g; iodine 1.0 g; zinc 13.3 g; selenium 0.2 g; antioxidants 26.6 g; excipients q.s.p. 2000.0 g. ^5^ Fugibam. ^6^ Butyl-hydroxy-toluene. ^7^ Gross energy based on values calculated for protein, 5.6 kcal g^−1^; lipid, 9.4 kcal g^−1^; carbohydrate, 4.1 kcal g^−1^ (NRC, 2011 [6]). ^8^ Carbohydrates (g kg^−1^) = 1000 (crude protein + ash + total lipids). ^9^ ∑SFA = saturated fatty acids, also including 15:0, 17:0, 20:0, 21:0, 22:0, 24:0. ^10^ ∑MUFA = monounsaturated fatty acids, also including 17:1n-7, 18:1n-7, 20:1n-9, 22:1n-11, 24:1n-9. ^11^ ∑PUFA = polyunsaturated fatty acids, also including 22:5n-3.

**Table 2:** Growth performance parameters and biometric index of juvenile *Arapaima gigas* fed diets with increasing levels of lipids for 60 days.

Parameters	% Dietary Lipids (DL)					One-Way ANOVA *p*-Value		Regression (Regr.)			
	6DL	10DL	14DL	18DL	22DL		*p*-Value	R^2^	Regr.	Opt. DL	Max. DL
S (%)	97.5a	98.8a	95.0a	82.5b	90.0b	0.003	0.006	0.31	L	-	-
FW (g)	389.1 ± 12.0a	375.7 ± 20.3a	313.9 ± 33.5b	211.6 ± 14.3c	174.9 ± 16.9c	<0.001	<0.001	0.90	SP	6.0	10.26
FL (cm)	37.9 ± 0.2a	36.8 ± 0.4ab	35.6 ± 1.0b	32.2 ± 0.6c	30.4 ± 0.9d	<0.001	<0.001	0.91	L	-	-
WG (g)	309.1 ± 12.0a	295.7 ± 20.3a	233.9 ± 33.5b	131.6 ± 14.3c	94.9 ± 16.9c	<0.001	<0.001	0.92	SP	6.0	10.26
LG (cm)	16.1 ± 0.2a	15.0 ± 0.4ab	13.8 ± 1.0b	10.3 ± 0.6c	8.5 ± 0.9d	<0.001	<0.001	0.91	L	-	-
FI (g)	618.1 ± 8.1a	618.1 ± 23.1a	565.8 ± 9.9b	540.5 ± 42.9b	526.2 ± 23.2b	<0.001	<0.001	0.69	SP	6.0	10.77
FCR	2.0 ± 0.1c	2.1 ± 0.2c	2.5 ± 0.4c	4.1 ± 0.4b	5.7 ± 0.9a	<0.001	<0.001	0.79	SP	6.0	10.62
PCR (%)	36.5 ± 0.5a	35.6 ± 1.5a	33.8 ± 2.1b	28.0 ± 0.9b	25.9 ± 1.1b	<0.001	<0.001	0.85	L	-	-
RGR (% day^−1^)	2.7 ± 0.1a	2.6 ± 0.1a	2.3 ± 0.1b	1.6 ± 0.1c	1.3 ± 0.2d	<0.001	<0.001	0.89	L	-	-
CF	0.7 ± 0.03a	0.8 ± 0.04a	0.7 ± 0.03a	0.6 ± 0.02b	0.6 ± 0.01b	<0.001	<0.001	0.58	L	-	-
HIS	1.5 ± 0.1	1.7 ± 0.1	1.6 ± 0.3	1.5 ± 0.1	1.4 ± 0.2	0.164	0.297	-	-	-	-
LSI	0.05 ± 0.03	0.07 ± 0.01	0.09 ± 0.05	0.09 ± 0.02	0.10 ± 0.02	0.154	0.015	0.25	L	-	-

Means were analyzed via one-way ANOVA and Tukey’s test, and different letters in the lines indicate statistical differences (*p* < 0.05). Results are shown as mean ± standard deviation (n = 4). S = survival. FW = final weight. FL = final length. WG = weight gain. LG = length gain. FI = feed intake. FCR = feed conversion rate. PCR = protein conversion rate. RGR = relative growth rate. CF = Fulton’s allometric condition factor. HSI = hepatosomatic index. LSI = lipo-somatic index. L = linear. SP = second-order polynomial. Opt DL = optimal lipid level to maximize that growth performance parameter. Max DL = maximum lipid level in the diet that does not hamper that parameter.

**Table 3:** Hematological and biochemical parameters of juvenile *Arapaima gigas* fed diets with increasing levels of lipids over a 60-day experimental period.

Parameters	% Dietary Lipids (DL)					One-Way ANOVA *p*-Value
	6DL	10DL	14DL	18DL	22DL	
Hematological parameters						
HEM (g dL^−1^)	7.8 ± 0.5	8.7 ± 1.1	7.1 ± 0.6	7.2 ± 2.4	8.8 ± 0.4	0.212
HT (%)	19.4 ± 1.93b	34.3 ± 8.9a	30.8 ± 4.8a	27.4 ± 5.8a	32.3 ± 2.2a	0.011
ERY (×10^6^ dL^−1^)	1.9 ± 0.3	2.8 ± 0.5	2.9 ± 0.6	2.4 ± 0.5	2.9 ± 0.5	0.051
MCHC (%)	40.1 ± 2.2a	26.1 ± 4.4b	26.7 ± 9.2b	25.7 ± 3.4b	26.8 ± 1.2b	0.006
MCV (fL)	102.0 ± 14.8	121.5 ± 26.4	104.9 ± 14.6	116.8 ± 25.9	114.6 ± 22.9	0.679
MCH (g dL^−1^)	40.6 ± 4.1	31.4 ± 6.4	28.0 ± 10.4	30.2 ± 8.4	31.1 ± 4.8	0.176
LEU (×10^5^ µL^−1^)	0.1 ± 0.01	0.1 ± 0.02	0.1 ± 0.02	0.1 ± 0.02	0.1 ± 0.03	0.054
NEU (%)	78.8 ± 10.7	84.0 ± 5.7	71.8 ± 3.8	84.5 ± 7.5	82.8 ± 8.0	0.144
LYM (%)	21.3 ± 10.7	16.0 ± 5.7	25.8 ± 8.6	15.5 ± 7.5	17.3 ± 8.0	0.393
Biochemical parameters						
GLU (mg dL^−1^)	32.3 ± 5.7a	44.3 ± 9.8a	36.0 ± 5.5a	23.3 ± 3.2b	22.5 ± 3.1b	0.042
TRI (mg dL^−1^)	48.3 ± 17.5	33.5 ± 8.4	53.3 ± 15.2	35.0 ± 11.1	44.0 ± 23.6	0.385
CLS (mg dL^−1^)	72.0 ± 13.7	72.8 ± 19.1	74.8 ± 16.1	58.3 ± 28.9	62.3 ± 7.5	0.114
PRO (g dL^−1^)	1.8 ± 0.2ab	2.2 ± 0.4a	2.1 ± 0.2a	1.5 ± 0.1b	1.8 ± 0.3ab	0.018

Means were analyzed via one-way ANOVA and Tukey’s test, and different letters in the lines indicate statistical differences (*p* < 0.05). Results are shown as mean ± standard deviation (n = 4). HEM = hemoglobin. HT = hematocrit. ERY = erythrocytes. MCHC = mean corpuscular hemoglobin concentration. MCV = mean corpuscular volume. MCH = mean corpuscular hemoglobin. LEU = leukocytes. NEU = neutrophils. LYM = lymphocytes. GLU = glucose. TRI = triglycerides. CLS = cholesterol. PRO = total protein.

**Table 4:** Proximate composition of the whole body and muscle of juvenile *Arapaima gigas* fed diets with increasing levels of lipids over a 60-day experimental period.

Parameters (g kg^−1^)	% Dietary Lipids (DL)					One-Way ANOVA *p*-Value
	6DL	10DL	14DL	18DL	22DL	
Whole body						
Moisture	744.9 ± 17.6	742.1 ± 16.6	743.4 ± 14.8	743.5 ± 18.5	745.4 ± 17.8	0.850
Crude Protein	149.1 ± 7.5	149.5 ± 7.6	150.6 ± 9.0	152.6 ± 6.1	150.0 ± 4.9	0.081
Lipids	44.6 ± 1.8a	45.1 ± 1.7a	41.5 ± 0.5b	40.4 ± 1.5b	41.6 ± 0.8b	<0.001
Ash	42.2 ± 1.5	42.5 ± 1.5	43.7 ± 1.6	43.0 ± 1.5	42.4 ± 1.6	0.054
Muscle						
Moisture	771.0 ± 16.8	776.6 ± 19.4	771.5 ± 17.5	778.2 ± 12.0	778.5 ± 18.5	0.430
Crude Protein	152.4 ± 5.1	154.7 ± 6.7	151.8 ± 4.6	153.7 ± 7.5	151.4 ± 6.6	0.120
Lipids	32.4 ± 2.3a	28.5 ± 2.5a	32.8 ± 2.0a	23.3 ± 2.8b	24.1 ± 1.7b	<0.001
Ash	30.2 ± 1.1	29.0 ± 1.5	29.4 ± 2.8	30.4 ± 1.4	32.1 ± 1.6	0.061

Means were analyzed via one-way ANOVA and Tukey’s test, and different letters in the lines indicate statistical differences (*p* < 0.05). Results are shown as mean ± standard deviation (n = 4).

**Table 5:** Fatty acid composition (mg g of total lipids^−1^) of the muscle of juvenile *Arapaima gigas* fed diets with increasing levels of lipids over a 60-day experimental period.

Parameters	% Dietary Lipids (DL)					One-Way ANOVA *p*-Value
	6DL	10DL	14DL	18DL	22DL	
∑SFA	281.8 ± 2.9a	267.5 ± 1.6b	260.5 ± 0.8c	262.2 ± 1.0c	257.7 ± 1.7d	<0.001
14:0	16.2 ± 0.2a	14.2 ± 0.1b	10.2 ± 0.4c	10.5 ± 0.1c	9.7 ± 0.1d	<0.001
16:0	164.0 ± 2.3a	154.0 ± 1.1b	152.3 ± 0.6c	159.0 ± 0.6c	149.3 ± 0.9d	<0.001
18:0	89.6 ± 2.9	87.6 ± 3.5	86.1 ± 3.3	85.1 ± 2.3	88.3 ± 6.7	0.080
∑MUFA	227.4 ± 1.0a	216.3 ± 1.1b	209.1 ± 1.0c	212.6 ± 2.7c	208.7 ± 0.9c	<0.001
16:1n-7	20.6 ± 1.4a	17.0 ± 0.4b	13.4 ± 0.2c	14.0 ± 0.2c	13.3 ± 0.1c	<0.001
18:1n-9c	154.0 ± 2.6a	143.0 ± 2.8b	135.7 ± 4.5c	138.6 ± 1.8c	134.7 ± 4.8c	<0.001
∑PUFA	432.2 ± 7.8c	449.8 ± 6.1b	455.8 ± 4.6b	448.0 ± 6.6b	466.4 ± 7.9a	<0.001
18:2n-6	169.9 ± 7.8a	148.4 ± 6.9b	107.5 ± 9.0c	110.4 ± 8.4c	152.3 ± 8.7b	<0.001
18:3n-3	12.6 ± 0.1a	11.4 ± 0.2b	8.0 ± 0.1c	8.0 ± 0.2c	10.9 ± 0.9b	<0.001
20:4n-6	4.2 ± 0.2a	3.7 ± 0.3ab	3.4 ± 0.1b	3.4 ± 0.3b	3.3 ± 0.1b	<0.001
20:5n-3	46.0 ± 0.4d	52.5 ± 0.4c	54.9 ± 0.5b	56.6 ± 0.7a	56.3 ± 0.5a	<0.001
22:6n-3	134.5 ± 0.4c	164.4 ± 1.0b	204.7 ± 0.5a	192.8 ± 1.4a	166.3 ± 2.2b	<0.001
∑n-3	242.4 ± 4.8c	284.1 ± 6.0b	331.5 ± 1.3a	331.5 ± 2.1a	296.6 ± 8.1b	<0.001
∑n-6	189.8 ± 3.3a	165.7 ± 4.7b	124.3 ± 6.9c	126.7 ± 5.0c	169.9 ± 5.8b	<0.001
n-3:n-6	1.3 ± 0.01c	1.7 ± 0.01b	2.7 ± 0.02a	2.5 ± 0.03a	1.8 ± 0.02b	<0.001

Means were analyzed via one-way ANOVA and Tukey’s test, and different letters in the lines indicate statistical differences (*p* < 0.05). Results are shown as mean ± standard deviation (n = 4). ∑SFA = saturated fatty acids, also including 15:0, 17:0, 20:0. ∑MUFA = monounsaturated fatty acids, also including 17:1n-7, 18:1n-7, 20:1n-9. ∑PUFA = polyunsaturated fatty acids, also including 18:3n-6, 18:4n-3, 20:3n-6, 20:3n-3, 22:5n-3.

## Data Availability

Data from this study are available from the corresponding authors upon reasonable request.
